# Altered monocyte activation markers in Tourette’s syndrome: a case–control study

**DOI:** 10.1186/1471-244X-12-29

**Published:** 2012-05-18

**Authors:** Judith Matz, Daniela L Krause, Sandra Dehning, Michael Riedel, Rudolf Gruber, Markus J Schwarz, Norbert Müller

**Affiliations:** 1 Department of Psychiatry and Psychotherapy, Ludwig Maximilian University, Nussbaumstr. 7, 80336, Munich, Germany; 2 Vinzenz von Paul Hospital, Psychiatry, Schwenninger Str. 55, 78628, Rottweil, Germany; 3 Department of Rheumatology, Ludwig Maximilian University, Pettenkoferstr. 8a, 80336, Munich, Germany

## Abstract

**Background:**

Infections and immunological processes are likely to be involved in the pathogenesis of Tourette’s syndrome (TS). To determine possible common underlying immunological mechanisms, we focused on innate immunity and studied markers of inflammation, monocytes, and monocyte-derived cytokines.

**Methods:**

In a cross-sectional study, we used current methods to determine the number of monocytes and levels of C-reactive protein (CRP) in 46 children, adolescents, and adult patients suffering from TS and in 43 healthy controls matched for age and sex. Tumor necrosis factor alpha (TNF-alpha), interleukin 6 (IL-6), soluble CD14 (sCD14), IL1-receptor antagonist (IL1-ra), and serum neopterin were detected by immunoassays.

**Results:**

We found that CRP and neopterin levels and the number of monocytes were significantly higher in TS patients than in healthy controls. Serum concentrations of TNF-alpha, sIL1-ra, and sCD14 were significantly lower in TS patients. All measured values were within normal ranges and often close to detection limits.

**Conclusions:**

The present results point to a monocyte dysregulation in TS. This possible dysbalance in innate immunity could predispose to infections or autoimmune reactions.

## Background

Tourette`s syndrome (TS) is a neuropsychiatric disorder that is characterized by childhood onset of multiple motor tics and at least one vocal tic. The disease shows exacerbations and remissions over time. Obsessive-compulsive disorder (OCD), attention deficit hyperactivity disorder (ADHD), mood disorders, and behavioral problems are often associated with TS [1]. TS can lead to social impairment, psychological distress, and discrimination; extreme forms of this disorder include self-injurious motor tics and coprolalia. Epidemiological studies show that TS is relatively common, with an estimated overall worldwide prevalence of 1% [2] and an even higher prevalence among children. Boys are affected about 4 times more often than girls [3]. The results of different studies point to a multifactorial pathogenesis of TS, with an interaction of genetic, environmental, hormonal, and immunological factors [4]. The exact pathogenic mechanisms are still unclear, but a disturbed dopaminergic neurotransmission in the basal ganglia-striato-thalamo-cortical circuits seems to generate tic expression as a common endpoint. Genetics play also an important role in the disease etiology of TS. However, TS is likely to be genetically heterogeneous and until now no loci have been definitely confirmed. Twin studies support a significant role for non-genetic influences [5].

One important hypothesis is that infections could trigger the onset of TS or tic exacerbations. Signs of acute or chronic infections have been observed in TS patients [6]. As both acute infections with A beta-hemolytic streptococcus (GABHS) [7,8] and poststreptococcal inflammation [9] have been reported to be associated with TS, TS has been proposed to be one of the pediatric autoimmune neuropsychiatric disorders associated with streptococcal infection (PANDAS) [10,11], similar to the pathophysiological model of Sydenham’s chorea. Moreover, increased streptococcal antibodies and signs of inflammation have been observed in adult TS patients [12,13]. Nevertheless, the topic of PANDAS is contentious, and it is not clear whether it is a distinct clinical entity [14]. Also, infections with Borellia burgdorferi [15], acute or chronic infections with Mycoplasma pneumoniae [12,13], and viral infections [16] have been reported to trigger tic exacerbations. Recently, we could report significantly more positive IgG antibody titers against Chlamydia trachomatis in TS patients than in healthy controls and a trend to higher antibody titers of Toxoplasma gondii in TS patients [17]. Both pathogens can invade the brain parenchyma. The role of autoantibodies against neuronal structures in TS is still controversial, and the causality is not proven; however, autoantibodies against neuronal structures could be one possible pathophysiological model for a subgroup of TS patients.

Another important hypothesis is the existence of a common underlying altered immune status that results in an insufficient clearing of infectious agents and triggers the onset of TS or worsening of tics. The innate immune system in particular is important for the first immune response. For this reason, we investigated monocytes; the mainly monocyte-derived cytokines tumor necrosis factor alpha (TNF-alpha), interleukin-6 (IL-6), and interleukin-1 receptor-antagonist (IL1-ra); and monocyte-derived soluble CD14 (sCD14). Cells of the monocyte/macrophage lineage phagocytize foreign material, present antigens to T-cells and produce proinflammatory cytokines, such as TNF-alpha, IL-1, and IL-6. Peripheral cytokines are known to be able to cross the blood–brain barrier and influence neuronal brain functions, even in low concentrations [18]. To our knowledge, so far no studies have focused mainly on monocytes and monocyte-derived parameters in TS and only a few studies have investigated the role of cytokines in TS. Leckman et al. [19] reported increased serum levels of TNF-alpha and IL-12 in juvenile TS patients. This is an interesting finding, because in OCD decreased levels of TNF-alpha were described [20-22]. Since OCD and TS show a high rate of co-morbidity [23], it would be of interest to find a possible discriminative marker (e.g. cytokines that are decreased in OCD and increased in TS) for the two disorders. Gabbay et al. investigated juvenile TS patients and reported higher IL-12 plasma levels in TS/OCD patients than in controls and higher IL-2 in the subgroup of TS/OCD patients than in TS patients without OCD [24]. Bos-Veneman et al. [25] did not evaluate findings of an immune activation or differences between TS and controls, but did find that serum IL-2 concentrations were positively associated with tic severity ratings.

Soluble CD14 plays an important role in innate immunity. Monocytes carry the surface marker CD14, a membrane-bound or soluble glycoprotein that mediates the interaction of lipopolysaccaride (LPS; a component of the cell surface of gram-negative bacteria) and of lipoteichoic acid (LTA; a component of different gram-positive bacteria) with cells. Mycoplasma pneumoniae can also be recognized by LPS-binding protein [26]. Soluble CD14 additionally enhances the immune response to LPS in cell types that lack the CD14 receptor on their surface [26], i.e. sCD14 is a co-activator for many cell types in the cascade of immune activation in response to a monocytic signal [27,28]. Decreased sCD14 levels point to a decreased state of monocyte activation. It has been postulated that sCD14 is important for the development of the T cell subset balance [29]. Reduced sCD14 levels in breast milk have been reported to be associated with the occurrence of atrophy and eczema [30]. So far, sCD14 has not been investigated in TS patients. Recently, reduced natural regulatory T (Tregs) cells in children with TS or OCD or both were reported, indicating a predisposition for overriding autoimmune responses [31].

To detect a possible (chronic) inflammatory state in TS patients, we measured neopterin and C-reactive protein (CRP), the most common and sensitive markers for an inflammatory process.

The study focused on innate immunity, because disturbances in innate immunity could explain an increased susceptibility to infectious agents.

## Methods

In a cross-sectional study performed at the Department of Psychiatry and Psychotherapy, Ludwig Maximilian University (LMU), Munich, Germany, we examined 46 TS patients and 43 age- and sex-matched healthy controls. All 46 TS patients fulfilled the DSM-IV-TR diagnostic criteria [32] for TS. Diagnoses were made by two independent experienced psychiatrists. Tic severity was quantified with the Tourette’s Syndrome Global Scale (TSGS) [33], and co-morbid OCD with the Maudsley Obsessive-Compulsive Inventory (MOCI) [34]. As a cut-off value for the group with OCD, study participants with a MOCI value of ≥ 12 were chosen (maximum value 30 points) [35]. The mean duration of TS was 21.1 years. The 43 healthy controls were recruited from the general population in Munich by advertisements. An experienced psychiatrist performed structured clinical interviews to exclude any psychiatric disorders. Exclusion criteria for patients and healthy controls were acute infections and immunological, neurological, or other medical disorders and were checked by assessing the medical history, clinical examination, and laboratory measurements (CRP, differential blood count, basic parameters of clinical chemistry). No treatment with corticosteroids or other immune-modulating agents was allowed. All study participants were Caucasians. Written informed consent was obtained from all study participants. If study participants were younger than 18 years, at least one parent had to give informed consent. The study was approved by the ethics committee of the LMU medical faculty.

A total of 18 ml blood was drawn from each participant: 3 ml EDTA blood was used for the differential blood count; 7.5 ml heparin blood, to determine CRP and basic clinical chemistry parameters (electrolytes, liver and kidney parameters, TSH); and 7.5 ml whole blood, to extract serum for later immunoassays. Within 30 minutes after vein puncture, 7.5 ml whole human blood was centrifuged at 3500 rpm at 4°C for 10 minutes and the extracted serum was aliquoted and stored at −80°C for later measurements. The differential blood count included a monocyte count and was performed on a hematology analyzer (Coulter STKS). CRP and basic clinical chemistry parameters were measured on an automated analyzer (ROCHE, Hitachi 912). To detect TNF-alpha and IL-6, we used the Bio-Plex cytokine assay and the cytokine reagent and diluent kit (Bio-Rad, Munich). The Bio-Plex system combines the principle of a sandwich immunoassay with Luminex fluorescent bead technology. The lower limits of detection were 0.2 pg/ml for TNF-alpha and 1.7 pg/ml for IL-6. sCD14 was detected in serum by using the Quantikine soluble CD14 Immunoassay (R&D Systems, Minneapolis), with a lower limit of detection of 0.25 ng/ml. Neopterin was measured with the neopterin solid phase enzyme-linked immunosorbent assay (IBL, Hamburg); the lower detection limit was 1.35 nmol/l . For IL1-ra, we used the Quantikine IL1-ra Immunoassay (R&D Systems, Minneapolis) with a lower limit of detection of 0.047 ng/ml. All assays were done according to the respective manufacturer’s instructions for use.

We used the SPSS 13.0.1. statistic program for statistical analysis. Data are given in means ± standard deviation (SD). Since some data did not pass the Kolmogorov-Smirnov test for normality, Mann–Whitney U tests were used to test differences between the patient and healthy control groups. P-values below alpha ≤ 0.05 were considered significant, and all tests were two-tailed. As the analyses were exploratory in nature and the monocyte-derived products seemed to be highly correlated, we did not correct for multiple tests. Results have to be interpreted accordingly. In addition, the results were analyzed by Kruskal-Wallis ANOVA and alpha-corrected post hoc Mann–Whitney U tests. Pearson’s correlation coefficients were calculated to test for possible correlations, and proportional data were analyzed by using Fisher’s exact test to test for homogeneity in the two groups.

## Results

### Study demographics

We found no significant differences between the two groups concerning age (χ_(2)_^2^ = 1.16, p = 0.60) or gender (χ_(1)_^2^ = 0.83, p = 0.47). Demographic data are summarized in Table 1.

**Table 1:** Demographic features and clinical characteristics of TS patients and healthy controls

	**n**	**TS patients**	**n**	**Healthy controls**
Age, mean ± SD (range)	**46**	**29.3 ± 14.1 (8–62)**	**43**	**30.6 ± 13.8 (8–62)**
< 18 years, n (%)	**15**	**33%**	**10**	**23%**
Age of onset, mean ± SD (range)	**46**	**8.2 ± 3.7 (2–17)**	**43**	**n.a.**
Male:female ratio (n)	**46**	**3.6:1 (36 m, 10 f)**	**43**	**2.3:1 (30 m, 13 f)**
TSGS values, mean ± SD (range)	**46**	**40.2 ± 13.0 (16.8 - 64.3)**	**43**	**n.a.**
OCD, n (%), MOCI ≥12, mean ± SD (range)	**34**	**74%** **13.1 ± 2.8 (12–26)**	**0**	**0%**
No OCD n (%), MOCI < 12 mean ± SD (range)	**12**	**26%** **0.42 ± 0.79 (0–2)**	**43**	**100%** **1.42 ± 0.96 (0–4)**
Psychotropic medication^1^, n (%)	**30**	**65%**	**0**	**0%**
Not medicated	**16**	**35%**	**43**	**100%**

TS = Tourette’s syndrome, OCD = obsessive compulsive disorder, MOCI = Maudsley Obsessive-Compulsive Inventory, TSGS = Tourette’s Syndrome Global Scale, n = number, f = female, m = male, SD = standard deviation, n.a. = not applicable.

^1^including neuroleptics (n = 18), tiapride (n = 11) and antidepressants (n = 7).

The medical history and basic clinical chemistry parameters showed no signs of any serious diseases, including hypo- or hyperthyreosis or acute infections, in either TS patients or healthy controls.

### Immune markers in Tourette’s syndrome

The mean number of monocytes/nl blood, CRP levels, and serum neopterin concentration were all significantly higher in TS patients than in healthy controls. However, these levels were within normal ranges in both groups. In contrast, the mean serum TNF-alpha concentration was significantly lower in TS patients than in healthy controls. IL-6 serum levels showed no differences between groups. Mean serum concentrations of sCD14 and mean IL1-ra concentrations were significantly lower in TS patients (see Table 2).

**Table 2:** Concentrations of immune parameters and monocyte markers in TS and healthy controls

**Variable**	**Group**	Mean ± SD	**Rank sum**	**z-value**	p-value*
**Monocytes/nl**	Patients	0.57 ± 0.22	50.1	−2.152	0.031
	Controls	0.47 ± 0.11	38.37		
**TNF-alpha pg/ml**	Patients	0.43 ± 0.18	33.09	−2.421	0.015
	Controls	0.56 ± 0.25	45.39		
**IL-6 pg/ml**	Patients	1.89 ± 1.71	39.41	−1.215	n.s.
	Controls	1,74 ± 1,64	33.43		
**IL1-ra ng/ml**	Patients	0.03 ± 0.09	36.11	−2.813	0.005
	Controls	0.04 ± 0.06	51.24		
**sCD14 ng/ml**	Patients	6.37 ± 1.43	30.25	−4.934	<0.0001
	Controls	8.40 ± 2.00	56,68		
**CRP mg/dl**	Patients	0.43 ± 0.27	45.53	−2.013	0.044
	Controls	0.37 ± 0.37	37.85		
**Neopterin nmol/l**	Patients	6.03 ± 2.19	48.51	−2.084	0.037
	Controls	5.19 ± 1.84	37.36		

SD = standard deviation, n.s. = not significant; *Mann–Whitney *U* Test.

Subgroup analyses of TNF-alpha, IL1-ra, sCD14, and neopterin did not reveal any differences between non-medicated and medicated TS patients. Similarly, no differences were seen between TS patients with and those without co-morbid OCD. Interestingly, the subgroup analyses revealed stronger differences in the comparison between TS patients with co-morbid OCD and healthy controls. These findings are shown in Table 3.

**Table 3:** Analysis of TNF-alpha, sCD14, IL1-ra, and neopterin in co-morbid OCD subgroups

		**TNF-alpha**	**sCD14**	**IL1-ra**	**Neopterin**
		**(pg/ml)**	**(ng/ml)**	**(ng/ml)**	**(nmol/l)**
**TS without OCD**	mean ± SD	0.52 ± 0.17	6.9 ± 1.3	0.013 ± 0.014	5.10 ± 1.28
**vs.**	rank sum	18.82	21.05	22.25	16.50
**TS with OCD**	mean ± SD	0.40 ± 0.18	6.3 ± 1.4	0.034 ± 0.095	6.36 ± 2.15
	rank sum	26.28	26.38	22.59	24.13
	p-value* (n)	n.s. (9/31)	n.s. (12/32)	n.s. (12/32)	n.s. (12/31)
**Control vs.**	mean ± SD	0.55 ± 0.24	8.4 ± 2,0	0.041 ± 0.057	5.16 ± 1.75
**TS with OCD**	rank sum	40.49	47.67	42.73	31.69
	mean ± SD	0.40 ± 0.18	6.3 ± 1.4	0.034 ± 0.095	6.36 ± 2.15
	rank sum	27.35	23.33	30.64	44.19
	p-value* (n)	**0.006** (37/31)	**0.000** (41/32)	**0.016** (42/32)	**0.013** (42/31)
**Control vs.**	mean ± SD	0.55 ± 0.24	8.4 ± 2.0	0.041 ± 0.057	5.16 ± 1.75
**TS without OCD**	rank sum	23.91	30.01	30.01	27.17
	mean ± SD	0.52 ± 0.17	6.9 ± 1.3	0.013 ± 0.014	5.10 ± 1.28
	rank sum	21.83	16.71	18.71	28.67
	p-value* (n)	n.s. (37/9)	**0.009** (41/12)	**0.028** (42/12)	n.s. (42/12)

TS = Tourette’s syndrome, OCD = obsessive compulsive disorder, n = number, SD = standard deviation, n.s. = not significant, *Mann–Whitney *U* Test.

Analyses of TNF-alpha, sCD14, IL1-ra, and neopterin for age subgroups are summarized in Table 4. Within the TS group, these parameters did not differ between children/adolescents and adults. Within the healthy control group, IL1-ra differed significantly between healthy children/adolescents and healthy adults; none of the other parameters differed between these two subgroups. Significant differences were found between healthy and TS children/adolescents for TNF-alpha, IL1-ra, and sCD14 and between healthy and TS adults for IL-1-ra, sCD14, and neopterin. Some of the subgroups were small, and the results have to be interpreted accordingly.

**Table 4:** Analysis of TNF-alpha, sCD14, IL1-ra, and neopterin in age subgroups

		**TNF-alpha**	**IL1-ra**	**sCD14**	**Neopterin**
		**(pg/ml)**	**(ng/ml)**	**(ng/ml)**	**(nmol/l)**
**Healthy**	mean ± SD	0.67 ± 0.24	0.056 ± 0.051	8.0 ± 1.4	4.70 ± 1.09
**children vs.**	rank sum	24.00	29.45	18.89	18.80
**healthy**	mean ± SD	0.51 ± 0.23	0.035 ± 0.058	8.6 ± 2.1	5.31 ± 1.90
**adults**	rank sum	17.15	19.02	21.59	22.34
	p-value* (n)	n.s. (10/27)	**0.017** (10/32)	n.s. (9/32)	n.s. (10/32)
**TS children**	mean ± SD	0.46 ± 0.20	0.056 ± 0.136	6.3 ± 1.0	5.35 ± 1.79
**vs.**	rank sum	22.50	25.07	20.37	17.54
**TS adults**	mean ± SD	0.41 ± 0.18	0.014 ± 0.096	6.6 ± 1.6	6.32 ± 2.07
	rank sum	19.42	21.17	23.60	24.16
	p-value* (n)	n.s. (14/26)	n.s. (15/29)	n.s. (15/29)	n.s. (14/29)
**Healthy**	mean ± SD	0.67 ± 0.24	0.056 ± 0.051	8.0 ± 1.4	4.70 ± 1.09
**children vs.**	rank sum	16.00	17.05	17.72	11.25
**TS children**	mean ± SD	0.46 ± 0.20	0.056 ± 0.136	6.3 ± 1.0	5.35 ± 1.79
	rank sum	10.00	10.30	9.37	13.39
	p-value* (n)	**0.042** (10/14)	**0.023** (10/15)	**0.003** (9/15)	n.s. (10/14)
**Healthy adults**	mean ± SD	0.51 ± 0.23	0.035 ± 0.058	8.6 ± 2.1	5.31 ± 1.90
**vs.**	rank sum	30.54	35.48	39.48	26.20
**TS adults**	mean ± SD	0.41 ± 0.18	0.014 ± 0.096	6.6 ± 1.6	6.32 ± 2.07
	rank sum	23.33	26.05	21.64	36.29
	p-value* (n)	n.s. (27/26)	**0.038** (32/29)	**0.000** (32/29)	**0.027** (32/29)

TS = Tourette’s syndrome, n = number, SD = standard deviation, n.s. = not significant, *Mann–Whitney *U* Test.

The neopterin concentration correlated significantly and positively with age in the patient group (r = 0.355, p = 0.02). None of the parameters correlated significantly with tic severity.

## Discussion

There is growing evidence that immunological mechanisms contribute to the pathophysiology of TS, at least in a subgroup of TS patients. We hypothesized that TS has a common underlying immune mechanism that possibly causes a disturbed clearing of infectious agents. As innate immunity plays an important role in the first-line immune response, in our study we focused on monocytes/macrophages and associated parameters.

Our results are in line with previous findings: Leckman et al. [19] reported signs of an increased activation of the cellular type-1 immune response in TS. At baseline, the authors found higher concentrations in TS patients than in controls of both the T cell (type 1) cytokine IL-12 and monocyte-derived TNF-alpha; the latter finding was in contrast to our findings. Even higher concentrations of these parameters were found in patients in a state of symptom exacerbation. Interestingly, these observations were more frequent in non-PANDAS than in PANDAS patients. Leckman et al. found no differences in the levels of other cytokines such as IL-1, IL-4, IL-6, IL-10, and IFN-γ [19]. In our sample, TNF-alpha serum levels were lower in TS patients than in healthy controls. Besides methodological differences, several factors could explain the differences between our findings. For example, our sample had a higher number of unmedicated patients. Our medicated patients received mainly dopamine antagonists, whereas patients in the study by Leckman et al. received mainly alpha-agonists. Certain antipsychotics are known to increase TNF-alpha levels [36], so our sample may have actually had even lower TNF-alpha levels if they had been in an unmedicated state. Although our sample size was limited, and the subgroups were particularly small, we did not find a significant influence of medication on any parameters, including TNF-alpha. Another contributing factor could be age [37] and disease stage. In our sample one limitation was the age range of study participants. This is why subanalyses were performed: healthy children/adolescents showed the highest serum TNF-alpha levels, followed by healthy adults, TS children/adolescents, and TS adults. When the subgroup of TS children/adolescents and TS adults were compared with the respective healthy control age groups, differences were seen between both age groups in almost all parameters. Given the high co-morbidity of OCD and TS [23], our results in regard to TNF-alpha levels are in accordance with reports of decreased TNF-alpha levels in OCD [20-22]. In our sample, the subgroup analysis of patients with and those without co-morbid OCD symptoms revealed no significant difference in the levels of any of the determined parameters, including TNF-alpha. This result indicates that in our sample the occurrence of co-morbid OCD seems to have no relevant influence on TNF-alpha levels. The same is true for other parameters, although the small size of the samples limits the interpretation of the findings. However, the trend of stronger effects in the comparison of healthy controls with TS patients with co-morbid ODC is noteworthy and in parallel with the findings of Gabbay et al. concerning IL-12 [24]. It has to be mentioned that in our study the subgroups of TS patients and TS patients with OCD could eventually suffer from a co-morbid ADHD. The structured clinical interviews did not show hints for ADHD in all study participants, however, in order to exclude ADHD entirely, a specific ADHD rating scale should be applied in future studies. Another limitation of this study is that the used rating scales (MOCI and TSGS) are reliable, but are less frequently used then for example the Yale-Brown Obsessive Compulsive Scale and do not represent the ‘gold standard’. In the present sample the mean value for OCD in the TS group was 13 out of a maximum of 30. Therefore these patients suffered only from a relatively mild OCD. More homogeneous patient samples and further studies are required to answer the question whether TS, TS with comorbid OCD, and OCD have to be seen as spectrum disorders with common causes or different pathophysiological mechanisms.

Since cytokines have to be seen as a network [38], TNF-alpha levels have to be discussed within the context of our other findings. Also, the decreased levels of IL1-ra suggest a decreased release of monocyte-derived cytokines and point to a decreased production of IL-1. Since IL-1 is released in a paracrine manner, the levels of circulating IL-1 in the blood often are below the limit of detection, are unstable and show a short half-life; therefore, the validity of estimating IL-1 serum levels is critically discussed. IL1-ra is a more stable and reliable laboratory parameter. It is produced and released from activated cells of the monocyte lineage [39]. No differences in IL-6 levels were detected between TS patients and healthy controls. IL-6 is assigned pro- and anti-inflammatory characteristics and plays a key role in the acute phase response, especially in direct resolutions of acute infections and septic shock. It is likely to play a detrimental role in chronic disease [40].

The most prominent finding—the lower levels of sCD14 in TS—could also reflect a decreased monocyte activation state and a possible susceptibility to infectious agents. The reduced sCD14 levels in TS patients may point to a blunted innate immunity and could indicate incomplete elimination of infectious agents and a chronic latent infectious state in TS. The most common and sensitive marker of inflammation is CRP. Although CRP levels were within the normal range in our sample of TS patients, the distinct but significant increase compared to controls could point to a latent underlying inflammatory process, similar to the role of high sensitivity CRP in the pathogenesis of atherosclerosis [41]. Neopterin, an unspecific marker of inflammation found in various inflammatory conditions such as infection and autoimmune syndromes [42], has been used as a measure of cell-mediated immunity [43]. The higher levels of neopterin in the TS group in our study reflects the activation of a Th-1 or type-1 immune response, since the type-1 cytokine Interferon-γ (INF-γ) induces neopterin production [44]. The findings of increased neopterin values in TS patients in our study are in line with previous findings. Hoekstra et al. reported significantly elevated neopterin levels in TS patients, indicating an immune activation [45].

In summary, our results point to changes in innate immunity in TS patients compared with healthy controls: sCD14 and the products of activated monocytes, TNF-alpha, and IL1-ra showed lower levels. The number of circulating monocytes, however, was increased. Whether this increase in monocytes might be a compensatory mechanism cannot be determined on the basis of the present data and needs to be investigated in future studies. These changes suggest a dysregulated immune system in TS, which could have implications for susceptibility to infections or autoimmunity.

In our study, not just one but several different parameters showed results pointing to the under-activation of components of the monocyte lineage associated with a possible subclinical inflammatory reaction. Moreover, the findings result from different laboratory methods, i.e. an immunoassay technique, the Luminex bead technology, and automated analyzer methods. As a caveat, it has to be noted that TNF-alpha, IL-6, IL1-ra, CRP, and neopterin serum levels were within the normal ranges and often close to the detection limits. Therefore, the increases or decreases in relation to the control group can only be interpreted as clues for potential trends. No functional tests were performed, and the data reflect only a single time point measurement of the peripheral immune system. Nevertheless, little is known about immunopathological mechanisms in TS, so that these preliminary data contribute to the understanding of monocyte regulation and innate immunity in TS.

To build a possible bridge between the peripheral immune system and the nervous system, one interesting fact is that microglia cells derive from the monocyte/macrophage lineage. Furthermore, cerebral hyperintensities in magnetic resonance images, which can be a sign of neuroinflammatory processes, seem to be associated with TS [46]. However, it remains unclear whether tic symptoms are partly due to the damage caused directly by infections or by the generated autoantibodies, or both, or due to changes in the immune balance. In both cases, neuronal signaling would be disturbed and could lead to tics. The central dopamine systems are most likely to play an important role in TS and can be modulated by immune function. In their review, Martino et al. [4] propose a pathophysiological model for immune-mediated dopamine synthesis via CaM kinase II activation. In this model, infections might induce cross-reactive anti-neural antibodies. First-line inflammatory responses might be promoted by infections and may potentiate dopamine release by autonomic fibers and modulate peripheral immune cells.

The immune constellation in our sample could be in accordance with an impaired innate immune response, associated with an imbalance of the specific (type-1 and type-2) immune responses, the type-2 responses possibly leading to infections, autoimmune phenomena, and inflammatory reactions. Nevertheless, one must take into account that TS is a heterogeneous syndrome that may have different underlying pathological mechanisms. The limited size of our sample—in particular with respect to interfering variables such as age, medication and co-morbidity—means that further intense research regarding the role of inflammation, infection, and the immune system in TS is required. Particularly functional tests and the analysis of genetic polymorphisms that encode for inflammatory cytokines or sCD14 would be of interest.

## Conclusions

The present findings point to monocyte dysregulation in TS. This possible dysbalance in innate immunity could lead to a higher susceptibility to infectious agents and a chronically latent inflammatory state in TS or could predispose to autoimmunity.

## Misc

†The authors JM and DLK contributed equally to this work

## Competing interests

The authors declare that they have no competing interests as regards this manuscript.

## Authors’ contributions

JM designed the study and recruited participants. DLK analyzed the results and prepared the manuscript, including a first draft. SD participated in the conception of the study and critically revised the manuscript. MR reviewed the included articles and assisted with the interpretation of the results. RG and MJK designed the study protocol and analyzed the laboratory parameters. NM revised the manuscript critically at each step of the analysis. All authors read and approved the final manuscript.

## Pre-publication history

The pre-publication history for this paper can be accessed here:

http://www.biomedcentral.com/1471-244X/12/29/prepub
